# High-Throughput and Untargeted Metabolic Profiling Revealed the Potential Effect and Mechanisms of Paeoniflorin in Young Asthmatic Rats

**DOI:** 10.3389/fphar.2022.829780

**Published:** 2022-02-08

**Authors:** Dan Wang, Li Zhao, Zhiyan Hao, Ying Huang, Yang Liao, Lingli Wang, Jinfeng Zhang, Shan Cao, Lixiao Liu

**Affiliations:** ^1^ Department of Pediatrics, Shanghai Pudong Hospital, Fudan University Pudong Medical Center, Shanghai, China; ^2^ Sanya Women and Children’s Hospital Managed by Shanghai Children’s Medical Center, Sanya, China

**Keywords:** metabolomics, metabolites, pathways, biomarker, action mechanism 3

## Abstract

Paeoniflorin (PF) is a multi-target monoterpenoid glycoside and possesses broad pharmacological functions, e.g., anti-inflammation, anti-depression, antitumor, abirritation, neuroprotection, antioxidant, and enhancing cognitive and learning ability. PF has gained a large amount of attention for its effect on asthma disease as the growth rate of asthma has increased in recent years. However, its mechanism of action on asthma is still unclear. In this study, we have explored the action mechanism of PF on asthma disease. Furthermore, high-throughput untargeted metabolic profiling was performed through ultraperformance liquid chromatography/electrospray ionization quadruple time-of-flight high-definition mass spectrometry (QA) UPLC-Q/TOF-MS combined with pattern recognition approaches and pathway analysis. A total of 20 potential biomarkers were discovered by UPLC/MS and urine metabolic profiling. The key pathways including the citrate cycle (the TCA cycle), pyrimidine metabolism, pentose phosphate pathway, tyrosine metabolism, and tryptophan metabolism were affected by PF. In conclusion, we have discovered metabolite biomarkers and revealed the therapeutic mechanism of PF based on liquid chromatography coupled with mass spectrometry untargeted metabolomics. The untargeted metabolomics combined with UPLC-MS is a useful tool for exploring the therapeutic mechanism and targets of PF in the treatment of asthma. Metabolomics combined with UPLC-MS is an integrated method to explore the metabolic mechanism of PF in the treatment of asthma rats and to reveal the potential targets, providing theoretical support for the study of the treatment of PF.

## Introduction

Asthma is characterized by chronic airway inflammation, airway hyperresponsiveness, and airway remodeling and is a serious public health problem in the world (Chiu et al., 2021). At present, the inhaled corticosteroids are the standard treatment for persistent asthma, but its antioxidant effect is not ideal (Koh et al., 2021; Werder et al., 2021). This treatment strategy has serious adverse reactions, such as headache, tremor, palpitations, and heart failure (Bush et al., 2021; Jin et al., 2021). In recent years, herbal medicine has received more and more attention due to its wide range of pharmacological effects, low toxicity, and few adverse reactions. Many herbal medicines and their active ingredients can effectively relieve asthma attacks and have become a hot spot for asthma treatment. Paeoniflorin (PF) is the major bioactive ingredient derived from *Paeonia lactiflora* Pall., *Paeonia suffruticosa* Andr., or *Paeonia veitchii* Lynch, which have been widely used for cardiovascular disease, cerebrovascular disease, and liver disease. PF is the main active ingredient of the commonly used *Paeonia lactiflora*. In recent years, it has been found that PF has anti-inflammatory, antitumor, antibacterial, antiviral, immune regulation, and scavenging free radicals and other pharmacological effects and has less toxic and side effects.

Paeoniflorin, a monoterpenoid glycoside, is derived from *Paeonia lactiflora* Pall., *Paeonia suffruticosa* Andr., or *Paeonia veitchii* Lynch which has been used in traditional medical applications for more than 2000 years. Recent works have showed that it has a wide range of pharmacologic activities, including anti-depression, anti-inflammatory, anti-oxidation, anti-apoptosis, antitumor, and maintaining mitochondrial function (Chen et al., 2021; Han et al., 2021). Interestingly, previous scientific evidences suggested that it possesses promising anticancer activities on diverse tumors/cancers (Yu et al., 2021; Zhao et al., 2021). Recently, growing attention has been paid to explore to relieve asthma attack function of PF (Jiao et al., 2021; Wu et al., 2021). It has been demonstrated to have potent anti-asthma activity in various types of mouse or rat models (Shou et al., 2019). Even though the concrete mechanisms are still not fully clarified, it is speculated to be associated with increasing the levels of monoaminergic neurotransmitters, inhibiting the overactivation of the HPA axis, promoting the neurogenesis and neuroplasticity, suppressing the neuroinflammation reaction, enhancing neuroprotection, etc (Fan et al., 2020; Wei et al., 2020; Liu et al., 2021). The numerous studies have focused on the in-depth mechanism and attempted to investigate the efficacy of PF in asthma treatment (Guo et al., 2021; Ma et al., 2021).

Metabolomics as an analytical strategy has been used to reveal the relationship between the chemical component and endogenous metabolic biomarkers (Sun et al., 2019; Fang et al., 2020; Qiu et al., 2020). In short, through monitoring metabolic trajectories and changes in metabolites caused by external factors *via* metabolomics (Liang et al., 2015a; Liang et al., 2015b), the natural product is used to characterize and identify *in vivo* metabolites to reveal the effect and mechanisms (Liang et al., 2016a; Liang et al., 2016b; Zhang et al., 2019a; Zhang et al., 2019b; Xie et al., 2019; Zhang et al., 2020). Furthermore, the potential molecular mechanisms corresponding to the anti-asthma effects of paeoniflorin are lacking which should be focused on further studies. In this study, ovalbumin (OVA) was used to prepare a mouse asthma model and explore the effect and mechanism of PF on an asthma rat. Our present work on anti-asthma effects of PF would be beneficial for the further molecular mechanism study of PF in the future.

## Methods and Materials

### Chemicals and Reagents

Paeoniflorin was supplied by *Chengdu PUSH Bio. Technology Co.*, Ltd. (batch number PS186203-01), and the HPLC chromatography is shown in Supplementary Material, Figure S1. *Wahaha* pure water was supplied by Hangzhou Wahaha Group Co., Ltd. Estradiol benzoate injection was supplied by Animal Pharmaceutical Hangzhou, China (batch number 11212511). Watson’s distilled water was supplied by Watson’s Food & Beverage Co., Ltd. (Guangzhou, China). Acetonitrile (HPLC grade) and methanol (HPLC grade) were supplied by Fisher (United States). Leucine enkephalin was supplied by Sigma-Aldrich (MO, United States). Other chemicals and reagents were of analytical purity.

### Animals

Wistar rats, 4 weeks old, weighing 85–100 g, were obtained from the Animal Center of Fudan University Pudong Medical Center. The room temperature and relative humidity were controlled at the range of 22–26°C and 35–45%, respectively, with a 12 h light/dark cycle. Prior to the experiment, all rats were put into the metabolism cages and allowed them to adapt to the environment for 7 days. Rats were given free access to water and normal food during this period. Then, we divided the rats into three groups stochastically: the control group (Con), the asthma model group (Mod), and the PF group (GY). The processes of the experiment were ratified by the Animal Care and Ethics Committee at the Fudan University Pudong Medical Center. In the light of the declaration of Helsinki, all experiments were carried out.

### Preparation of Juvenile Asthmatic Rats

The young rats were divided into three groups according to the random number method: the control group, the model group, and the PF group, each with 10 rats. According to the method in the literature (Han et al., 2017), a juvenile asthma rat model was prepared. Except the control group, the other groups were intraperitoneally injected with 0.2 ml OVA suspension (OVA 20 μg + 2 mg hydrogen) on days 1, 8, and 15. Alumina gel was sensitized, and the control group was injected with the same amount of normal saline. From the 16th day, the pups of the model group and treatment group were given a 4% OVA normal saline nebulized inhalation challenge once a day for 7 days. The pups in the control group were given the same amount of saline inhalation. After 1 hour, the pups of the control group and model group were intraperitoneally injected with normal saline, and the administration group was intraperitoneally injected with 9 mg/kg of PF.

### Urine Sample Preparation

The urine samples were collected and then centrifuged at 13,000 rpm/min in 4°C for 10 min. Before the UPLC-MS analysis, the supernatants were stored at −80°C. Before analysis, the urine samples were thawed in an ice bath; For analysis, 500 μL urine and 500 μL ultra-pure water were taken, vortexed for 30 s, centrifuged at 13,000 rpm for 15 min at 4°C, and filtered over 0.22 μm; the supernatant was injected into the UPLC-MS.

### Chromatography

The separation was accomplished through the ACQUITY UPLCTM phenomenex column (2.1 nm × 50 mm, 1.7 µm), controlling the column temperature at 40°C. The optimal mobile phase contained (A) acetonitrile with 0.1% formic acid and (B) water with 0.1% formic acid. The detailed chromatographic conditions are in Supplementary Table S1.

### Mass Spectrometry Analysis

High-resolution mass spectrometry was performed on a TOF MS/MS system (Waters Corporation, United States) equipped with an electrospray ionization (ESI) source in both positive- and negative-ion modes. The optimized conditions were as follows: positive mode parameters: ion source temperature 110°C, capillary voltage 3000 V, cone voltage30 V, extraction cone voltage 5.0 V, desolvation temperature 350°C, cone gas flow 50 L/h, and desolvation gas flow 800 L/h; negative mode parameters: capillary voltage 2800 V, cone voltage 40 V, and all other parameters the same as in the positive-ion mode.

### Biomarker Identification

The metabolic profiles (.raw data) obtained by using the UPLC-MS system were imported into Progenesis QI software (V2.1, Waters Corporation, MA) for alignment, peak picking, and normalization. Then, resultant data matrices (.usp data) were opened by Ezinfo software (V3.0) for multivariate statistical analysis through pattern recognition methods. Metabolic data matrices between the control group and PF group were analyzed by OPLS-DA. A preliminary VIP value was selected for primary screening of potential biomarkers. The secondary screening was carried out according to the principle that each metabolite in the control and model groups had a significant difference (*p* < 0.05) which could be considered to be the possible biomarkers. Then, the mol file and secondary mass spectrometry information were matched. Finally, the urine biomarkers of asthma were confirmed.

### Metabolic Pathway Analysis

The KEGG, HMDB, or the name of the compound of the detected biomarker was introduced to the website http://www.metabioanalyst.ca for metabolic pathway analyses. The metabolomic pathway related to metabolites was found by analyzing the topological characteristics of the pathway. The metabolic pathways related to the ECB model are obtained and mapped, and then, the schematic diagram of each metabolic pathway was obtained. The impact threshold was set to 0.10. Any pathway beyond this threshold was classified as a potential target pathway.

### Statistical Analysis

Data was conveyed by Student’s t-test and expressed as means ± SD. Differences in the average value were calculated statistically significant, and *p* < 0.05 was considered statistically significant and *p* < 0.01 meaning an extremely significant difference.

## Results

### Metabolic Profiling Analysis

**FIGURE 1:**
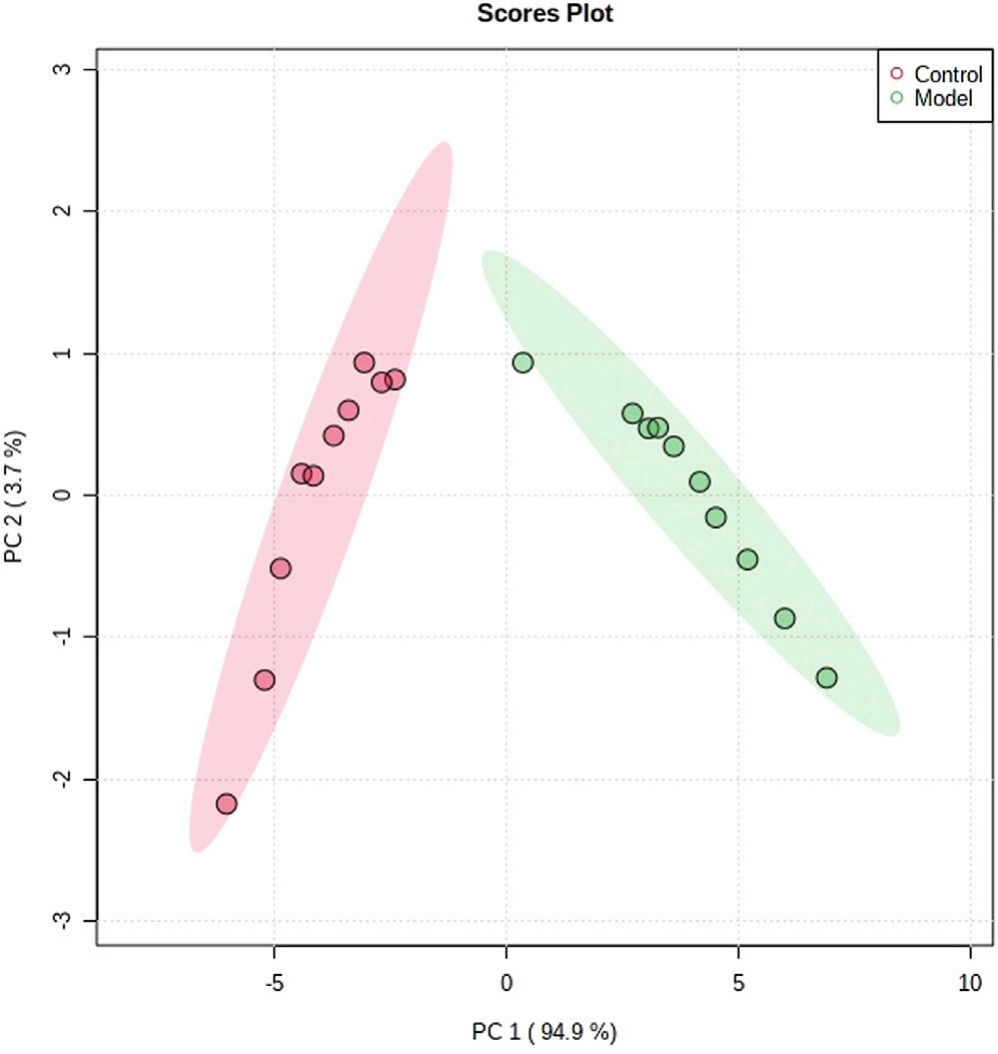
Metabolic profile characterization of PCA score plots for the control and model groups.

**FIGURE 2:**
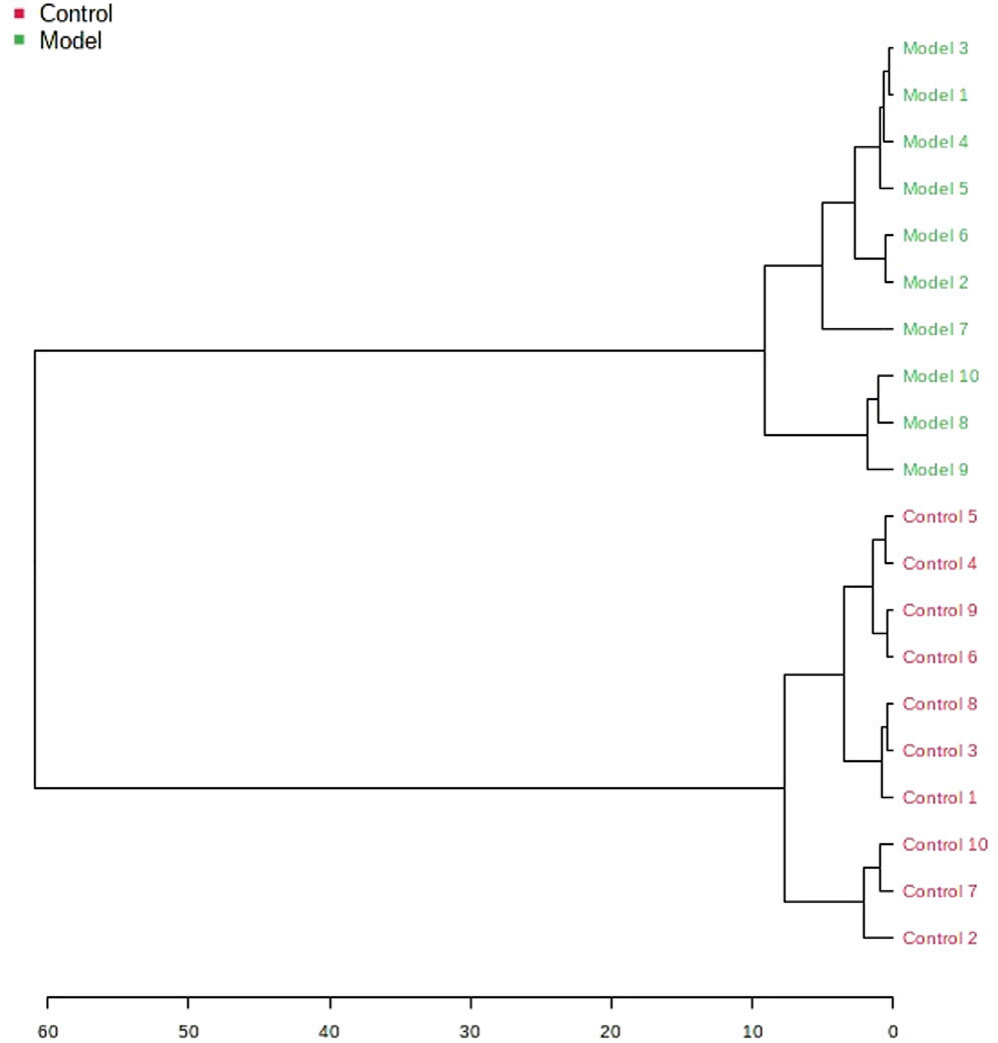
Hierarchical clustering dendrogram for the control and model groups.

**FIGURE 3:**
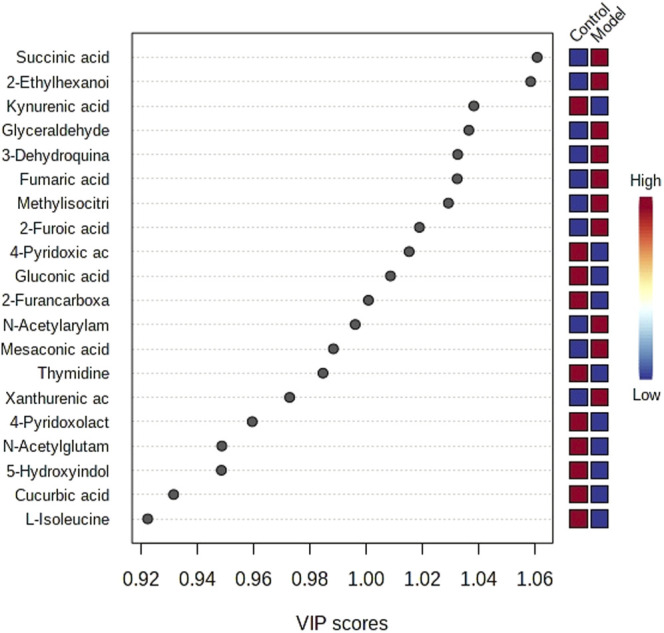
Top significant features of the metabolite markers based on the VIP projection.

The obtained UPLC-MS/MS urine metabolic spectrum data were entered into Progenesis QI for chromatographic peak alignment, data normalization, peak extraction, and multivariate statistical analysis. PCA of the data was performed by the MetaboAnalyst software module, and it was determined whether the model creation resulted in changes in endogenous components. The PCA pattern recognition was performed on the control group’s urine metabolic profile and the model group (Figure 1). PCA was conducted using urine sample data, and the final model revealed that the data profile of the model group was far away from that of the control group, which indicated a significant change in the metabolic network in the model group (Figure 1). To further distinguish the differences between different groups, hierarchical clustering dendrogram analysis was performed on urine metabolites in the two groups (Figure 2). There are clear differences between the control and model groups, indicating that the model group’s metabolism has changed after asthma. The VIP-plot is employed to screen potential biomarkers (Figure 3), the student’s t-test is applied to analyze the difference ions with VIP>1, and the normalized abundance is statistically significant (*p* < 0.05); these important difference ions are considered as potential biomarkers, combining MS and MS/MS structural information, including gluconic acid, 2-furoic acid, 2-ethylhexanoic acid, glyceraldehyde, cucurbic acid, N-acetylarylamine, and fumaric acid, and finally, these potential biomarkers were found to be closely related to asthma (Supplementary Table S2).

### Identification of Urine Biomarkers

**FIGURE 4:**
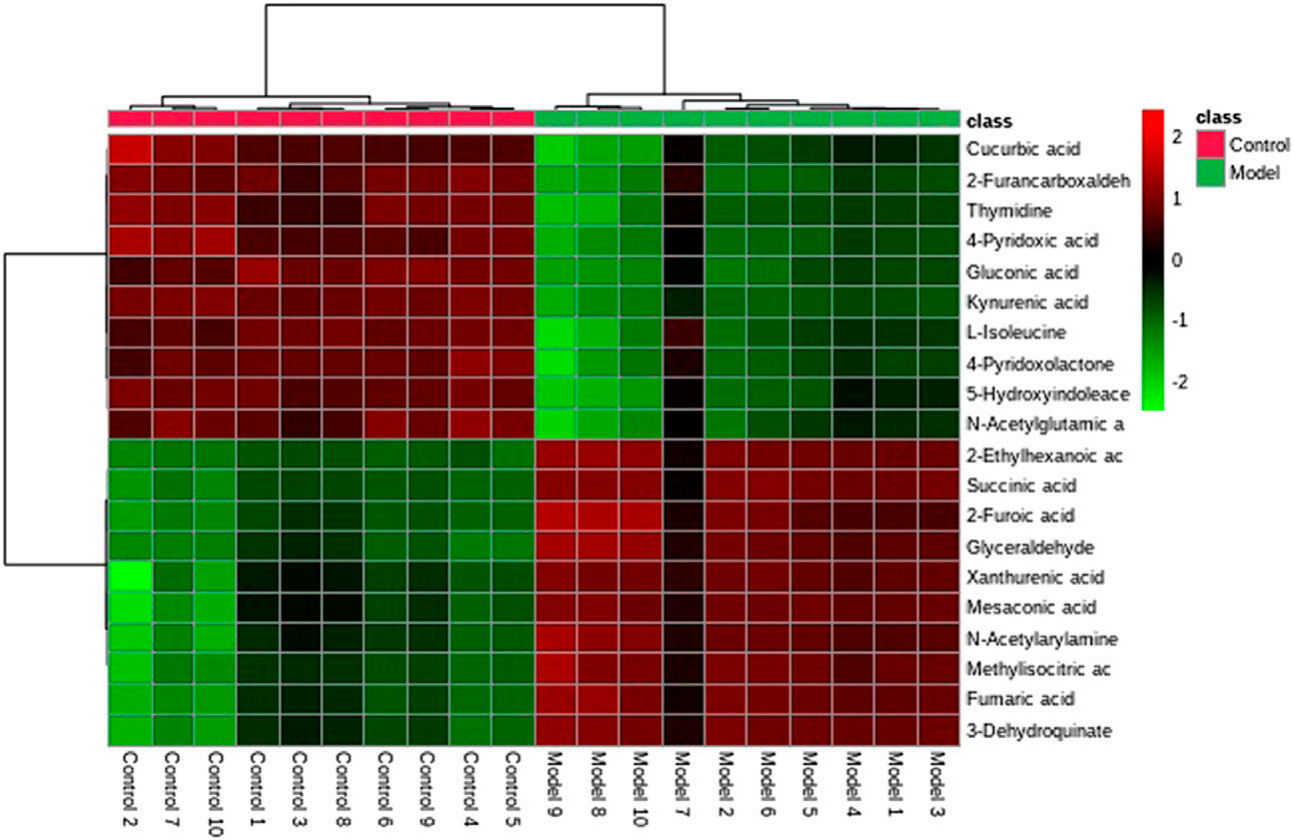
Hierarchical clustering of the differential metabolites in the control and model groups.

A UPLC-MS/MS high-throughput analyzer coupled with Progenesis QI was used to determine the precise molecular mass and to generate MS/MS data for the structural identification of biomarkers. MetaboAnalyst was used to analyze the blood metabolic profiles of rats in the model and control groups, and hierarchical clustering dendrogram diagrams, which directly reflect the contribution of each component to the change in the metabolic profile, were created (Figure 4) to highlight the maximum difference between groups. The selected components were identified by determining their relative molecular weight by primary MS and obtaining their structural fragment information. A total of 20 potential biomarkers were collected using multiple databases, including the HMDB, KEGG, and METLIN, and the expression levels of the potential biomarker in the control and model groups are showed in Figure 4.

### Metabolic Pathway Analysis

**FIGURE 5:**
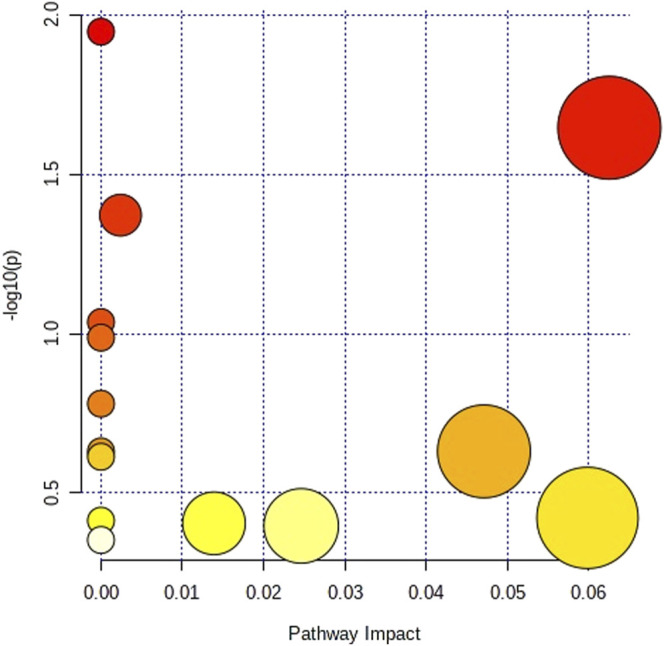
Metabolic pathway analysis with the MetaboAnalyst.

MetPA was used to construct and analyze metabolic pathways; the species was set to rat, and the HMDB numbers of the potential metabolites were entered for this pathway analysis. Using topological analysis, the cutoff value of the metabolic pathway influence was set to 0.01, and pathways with a value greater than 0.01 were selected as potential key metabolic pathways. A total of 5 metabolic pathways were identified as related to asthma, including the citrate cycle (TCA cycle), pyrimidine metabolism, pentose phosphate pathway, tyrosine metabolism, and tryptophan metabolism (Figure 5; Supplementary Table S3). Therefore, the above metabolic pathways were identified as target pathways. The results showed that these biomarkers are involved in multiple metabolic pathways which were closely related to the asthma model.

### Therapeutic Effect of PF on Asthma Model Rats

**FIGURE 6:**
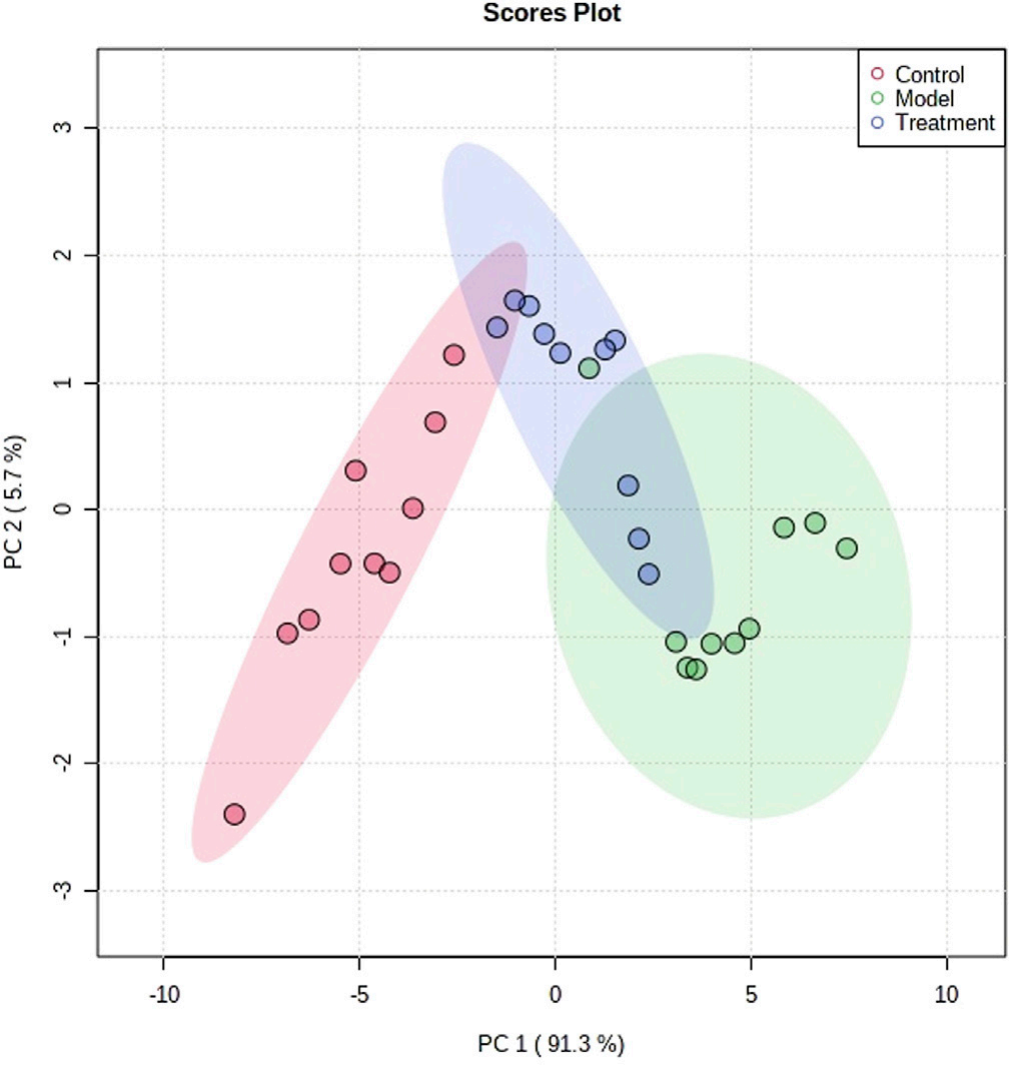
PCA score plots of multivariate data analysis.

**FIGURE 7:**
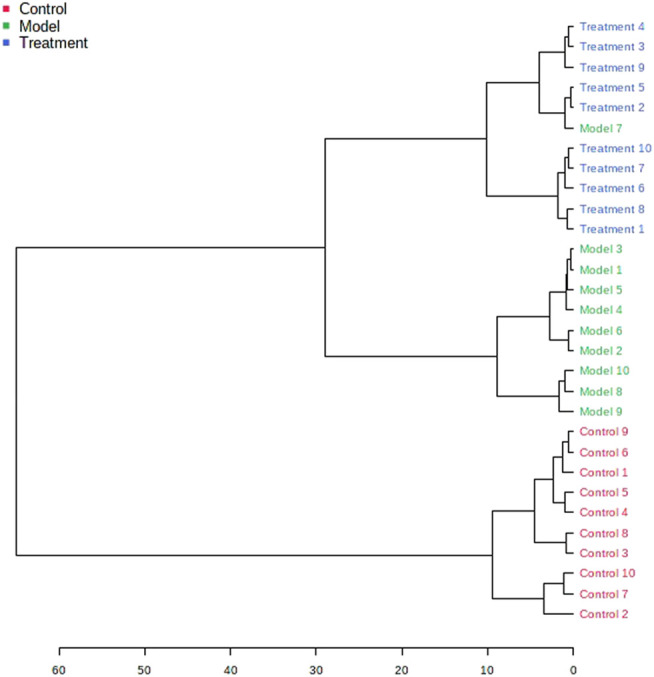
Hierarchical clustering dendrogram for the control, model, and treatment groups.

**FIGURE 8:**
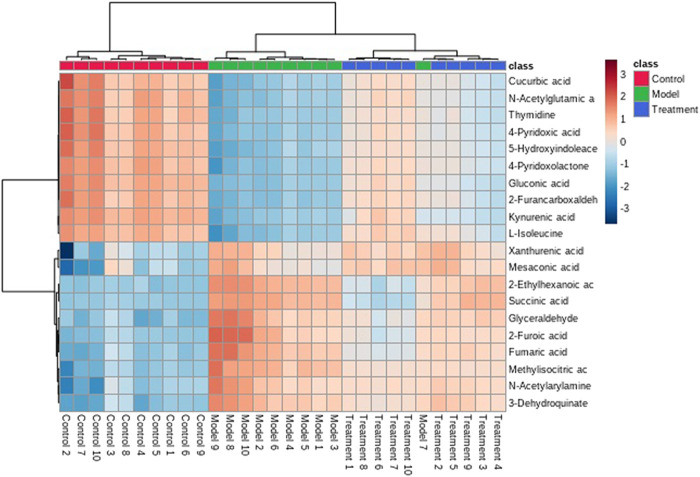
Cluster analysis of the differential metabolites in the control, model, and treatment groups.

The metabolic profile of urine at the last day of administration was input into the Progenesis QI software for processing. Subsequently, these ions were processed by MetaboAnalyst to obtain score plots (Figure 6) that can reflect the trend among groups. The control group and the asthma model group showed obvious clustering and separation. After the administration, the model rats had a tendency approach to the control group. By analyzing the trend of the urine biomarkers, we found that PF can affect the microbiological changes of potential biomarkers in model rats, and the content of these biomarkers tends to approach the control group (Figure 6). The spatial distribution of PCA scores revealed that rats in the model group could be obviously distinguished from those in the control group, indicating that endogenous regulation alone cannot normalize the metabolic changes in asthma rats to match metabolite levels in control rats after treatment. After the oral administration, the position of these treated rats was far from that of model rats and close to that of the control rats, which indicates that PF was able to normalize the asthma metabolic profile (Figure 7). PF can reverse the abnormal levels of these biomarkers, and the relative levels of biomarkers before and after treatment are shown in Figure 8.

## Discussion

Paeoniflorin is a pinane monoterpene glycoside with various bioactivities, such as anti-oxidative stress, anticancer effects, antiplatelet aggregation, and anti-inflammatory and reducing blood viscosity activity, and these pharmacological effects lay the foundation for PF of being a potential therapeutic agent for several diseases. Interestingly, the previous studies suggested that it possesses promising anticancer activities. Paeoniflorin has gained a large amount of attention for its effect on asthma disease as the growth rate of asthma has increased in recent years. This work will explore the related molecular mechanisms, which would be beneficial for the further exploration and development of this natural compound. However, the current investigations on action mechanisms of PF are lacking of *in vivo* experiments.

The high-throughput metabolomics could monitor the metabolites’ changes *in vivo* (Liang et al., 2016c; Liang et al., 2016d; Yu et al., 2017; Zhang et al., 2017; Kachroo et al., 2021). The current metabolomic analysis strategy is widely employed in the metabolic mechanism research and discovery of biomarkers (Zhang et al., 2015; Zhang et al., 2016; Liang et al., 2017).

In this study, UPLC-Q/TOF-MS was used to develop an untargeted metabolomics analysis of asthma rats to explore the overall metabolic changes and characterize biomarkers. As a result, a total of 20 biomarkers with VIP>1 and normalized abundance significant *p* < 0.05 were discovered. It is worth noting that PF reversed the levels of biomarker metabolites after treatment, which participated in 5 metabolic pathways including the citrate cycle (TCA cycle), pyrimidine metabolism, pentose phosphate pathway, tyrosine metabolism, and tryptophan metabolism. The biomarkers involved in these metabolic pathways play an important role in asthma. There are defects in this experiment. First of all, the current detection technology cannot analyze all the metabolites of the body at the same time and cannot reasonably interpret all the information obtained. It is necessary to further improve the metabolomic analysis detection technology and data processing technology. Further research is needed in terms of their use in clinical diagnosis.

## Conclusion

In this study, an advanced, high-sensitivity and high-throughput UPLC-MS was used to prove the PF-possessed therapeutic effects on asthma in some degree. This study explored the mechanism of PF in improving the rat model of asthma by metabolomics; a total of 20 potential biomarkers were identified in asthma rats. In addition, these biochemical indicators were regulated significantly after the treatment of PF, and five related pathways were significantly affected by PF. Our research had demonstrated that PF was effective against asthma. It has provided the scientific evidence for PF treatment for asthma disease.

## Data Availability

The original contributions presented in the study are included in the article/Supplementary Material; further inquiries can be directed to the corresponding authors.
