# Supplementary material for: High-Throughput and Untargeted Metabolic Profiling Revealed the Potential Effect and Mechanisms of Paeoniflorin in Young Asthmatic Rats
Source: Front Pharmacol. 2022 Feb 8;13:829780. doi: 10.3389/fphar.2022.829780 (PMC8861441; doi:10.3389/fphar.2022.829780)
Supplement: Supplementary file 1 [file DataSheet1.docx]

Table S1. The gradient elution program for UPLC analysis

| Time (min) | Flow rate (mL/min) | A% | B% |
| --- | --- | --- | --- |
| Initial | 0.400 | 2.0 | 98.0 |
| 5 | 0.400 | 40.0 | 60.0 |
| 9 | 0.400 | 70.0 | 30.0 |
| 12 | 0.400 | 100.0 | 0.0 |
| 14 | 0.400 | 100.0 | 0.0 |
| 15 | 0.400 | 100.0 | 0.0 |

Table S2. Serum biomarkers identiﬁed by high-throughput UPLC mass spectrometry.

| **No.** | **Rt (min)** | **m/z** | **ID** | **Adducts** | **Formula** | **ppm** | **Compound** |
| --- | --- | --- | --- | --- | --- | --- | --- |
| 1 | 6.97 | 195.0547 | HMDB00625 | M-H | C6H14O7 | 1.43 | Gluconic acid |
| 2 | 6.28 | 113.0095 | HMDB00617 | M+H | C5H4O3 | -3.93 | 2-Furoic acid |
| 3 | 6.17 | 143.1084 | HMDB31230 | M-H | C8H18O2 | -2.48 | 2-Ethylhexanoic acid |
| 4 | 6.08 | 89.0250 | HMDB01051 | M-H | C3H8O3 | -1.27 | Glyceraldehyde |
| 5 | 5.31 | 211.1334 | HMDB29388 | M-H | C12H22O3 | -1.43 | Cucurbic acid |
| 6 | 4.07 | 136.0620 | HMDB01250 | M+H | C8H9NO | -1.14 | N-Acetylarylamine |
| 7 | 3.71 | 117.0040 | HMDB00134 | M+H | C4H4O4 | -1.15 | Fumaric acid |
| 8 | 3.43 | 190.0543 | HMDB00763 | M-H | C10H11NO3 | 1.92 | 5-Hydroxyindoleacetic acid |
| 9 | 3.24 | 118.0357 | HMDB00715 | M-H | C10H9NO3 | -3.20 | Kynurenic acid |
| 10 | 2.84 | 130.0880 | HMDB00172 | M-H | C6H15NO2 | -2.93 | L-Isoleucine |
| 11 | 2.81 | 95.0144 | HMDB32914 | M-H | C5H6O2 | -1.38 | 2-Furancarboxaldehyde |
| 12 | 2.63 | 204.0295 | HMDB00881 | M-H | C10H9NO4 | -1.37 | Xanthurenic acid |
| 13 | 2.37 | 166.0361 | HMDB03454 | M+H | C8H7NO3 | -2.98 | 4-Pyridoxolactone |
| 14 | 2.36 | 241.0818 | HMDB00273 | M-H | C10H16N2O5 | -1.41 | Thymidine |
| 15 | 1.87 | 182.0466 | HMDB00017 | M-H | C8H11NO4 | -1.89 | 4-Pyridoxic acid |
| 16 | 1.51 | 119.0200 | HMDB00254 | M+H | C4H6O4 | -2.86 | Succinic acid |
| 17 | 1.48 | 207.0361 | HMDB06471 | M+H | C7H10O7 | -2.03 | Methylisocitric acid |
| 18 | 1.45 | 190.0570 | HMDB01138 | M+H | C7H11NO5 | -1.69 | N-Acetylglutamic acid |
| 19 | 1.37 | 189.0411 | HMDB12710 | M-H | C7H12O6 | -2.06 | 3-Dehydroquinate |
| 20 | 1.18 | 131.0203 | HMDB00749 | M+H | C5H6O4 | -1.12 | Mesaconic acid |

Table S3. Result from ingenuity pathway analysis with Metaboanalyst.

| **No.** | **Pathway name** | **Total** | **Expected** | **Hits** | **Raw p** | **Impact** |
| --- | --- | --- | --- | --- | --- | --- |
| 1 | Citrate cycle (TCA cycle) | 20 | 0.24 | 2 | 0.02 | 0.06 |
| 2 | Pyrimidine metabolism | 39 | 0.47 | 1 | 0.38 | 0.06 |
| 3 | Pentose phosphate pathway | 22 | 0.26 | 1 | 0.23 | 0.05 |
| 4 | Tyrosine metabolism | 42 | 0.50 | 1 | 0.40 | 0.02 |
| 5 | Tryptophan metabolism | 41 | 0.49 | 1 | 0.39 | 0.01 |
| 6 | Alanine, aspartate and  glutamate metabolism | 28 | 0.33 | 2 | 0.04 | 0 |
| 7 | Arginine biosynthesis | 14 | 0.17 | 2 | 0.01 | 0 |
| 8 | Valine, leucine and  isoleucine biosynthesis | 8 | 0.10 | 1 | 0.09 | 0 |
| 9 | Vitamin B6 metabolism | 9 | 0.11 | 1 | 0.10 | 0 |
| 10 | Butanoate metabolism | 15 | 0.18 | 1 | 0.17 | 0 |
| 11 | Pyruvate metabolism | 22 | 0.26 | 1 | 0.23 | 0 |
| 12 | Propanoate metabolism | 23 | 0.27 | 1 | 0.24 | 0 |
| 13 | Valine, leucine and  isoleucine degradation | 40 | 0.48 | 1 | 0.39 | 0 |
| 14 | Aminoacyl-tRNA biosynthesis | 48 | 0.57 | 1 | 0.44 | 0 |

Note: Total: The total number of compounds in the pathway; Hits: The number of accurate matching markers in the upload data; Raw p: Original P values obtained by pathway analysis; Impact: The affected value of pathways obtained by topological analysis.


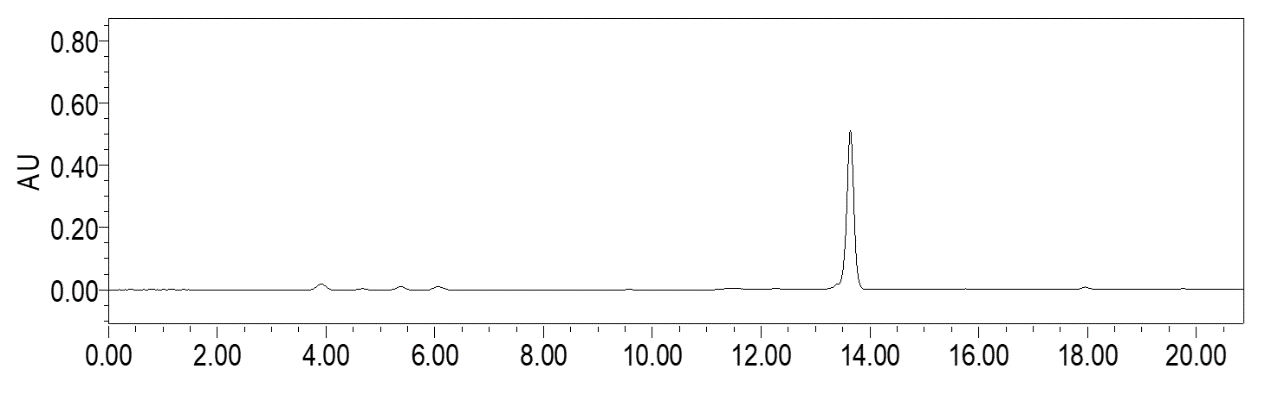


Figure S1. The HPLC method for paeoniflorin. Note: Paeoniflorin were separated on column Agilent ZOＲBAX SB-C18( 4.6×250mm, 5um) and detected at 230 nm, with acetonitrile-0.1% phosphoric acid solution as mobile phase. The flow rate was 1.0 mL·min-1.
